# Ultrastructural Features of Ischemic Tissue following Application of a Bio-Membrane Based Progenitor Cardiomyocyte Patch for Myocardial Infarction Repair

**DOI:** 10.1371/journal.pone.0107296

**Published:** 2014-10-13

**Authors:** Dehua Chang, Zhili Wen, Yuhua Wang, Wenfeng Cai, Mashhood Wani, Christian Paul, Teruo Okano, Ronald W. Millard, Yigang Wang

**Affiliations:** 1 Department of Advanced Biomedical Engineering and Science, Tokyo Women's Medical University, Tokyo, Japan; 2 Department of Pathology and Laboratory Medicine, University of Cincinnati, Cincinnati, Ohio, United States of America; 3 Department of Pharmacology and Cell Biophysics, University of Cincinnati, Cincinnati, Ohio, United States of America; Northwestern University, United States of America

## Abstract

**Background and Objective:**

Implantation of cell-sheets into damaged regions of the heart after myocardial infarction (MI) has been shown to improve heart function. However, the tissue morphology following application of induced pluripotent stem cell (iPSC)-derived cardiomyocytes (CM) has not been studied in detail at the level afforded by electron microscopy. We hypothesized that increasing the number of CM derived from iPSC would increase the effectiveness of cell-sheets used to treat ischemic cardiomyopathy. We report here on the ultrastructural features after application of a bio-membrane ‘cell patch’.

**Methods:**

iPSC-derived progenitor cells were transduced using lentivirus vectors with or without NCX1 promoter. iPSC-CM sheets were transplanted over the transmural MI region in a mouse model of regional ischemic cardiomyopathy. Mice were divided into four groups, 1) Sham; 2) MI; 3) MI + iPSC without NCX1 treated cells (MI + iPSC^Null^) and 4) MI + iPSC receiving NCX1 promoter treated cells (MI + iPSC^NCX1^). Echocardiography was performed 4 weeks after cell patch application, followed by histological and transmission electron microscopy (TEM) analysis.

**Results:**

Large numbers of transplanted CM were observed with significant improvements in left ventricular performance and remodeling in group 4 as compared with group 3. No teratoma formation was detected in any of the treatment groups.

**Conclusion:**

Manipulation of iPSC yields large numbers of iPSC-CM and favorable morphological and ultrastructural tissue changes. These changes have the potential to enhance current methods used for restoration of cardiac function after MI.

## Introduction

Despite recent advances in pharmacological and surgical approaches to rescue injured myocardium, ischemic heart disease remains the leading cause of heart failure and death [1]. Intravenous [2] or direct intramyocardial injections into an infarcted area [3] are the most common routes of cell delivery for myocardial therapy. It is difficult, however, to control the requisite and optimally targeted deposition of cells using these methods [4], [5]. Recent progress in myocardial cell sheet or cell patch techniques offers a potentially advantageous strategy for tissue engineering aimed at cardiac tissue regeneration by reversal of deleterious effects following myocardial infarction (MI).

Approaches using iPSC have gained credibility as an alternative treatment for infarcted myocardium repair in animal models. iPSC have also been used successfully as tools for drug development and modeling of diseases [6] [7]. iPSC possess the remarkable capacity to differentiate into a variety of cell lineages including CM, endothelial cells, and smooth muscle cells. They can also form teratomas, and if iPSC are to become a viable cell therapy option, it is of vital importance to minimize or eliminate the possibility of teratoma formation. This can be accomplished by directing iPSC *ex vivo* to differentiate exclusively into the desired cell types before transplantation.

Cell-sheet grafts are an attractive solution for providing large numbers of CM to the infarcted myocardium as a “progenitor cell reservoir.” More than 80% of cells transplanted using traditional cell injection techniques are either non-viable or absent within a first week after transplantation, and these injection methods and cells can cause acute inflammation and lethal arrhythmias [8]. Direct injection of cells into heart tissue is also hazardous due to potential blockage of microcirculatory pathways that can result in life-threatening complications [9]. Cell sheets, while requiring an invasive surgical procedure, achieve the desired goal of transplanting a large number specific progenitor cells into ischemic heart tissue. Such cell sheets also can strengthen the infarcted myocardial wall, reduce LV collagen deposition and prevent or reverse further LV remodeling [10]. Thus, we postulated that genetic manipulation of iPSC to assure a maximum amount of progenitor CM were present within a cell sheet could lead to an increase in subsequent differentiation and repopulation of functional CM within the infarcted area. If tissue regeneration due to the strategic enhancement of iPSC-derived CM was successful, we expected to observe beneficial changes in tissue morphology and ultrastructure of the MI region and an accompanying improvement in contractility of the left ventricle.

## Materials and Methods

### Laboratory animals

All research protocols conformed to the Guidelines for the Care and Use of Laboratory Animals published by the National Institutes of Health (National Academies Press, 8^th^ edition, 2011). All animal use protocols and methods of euthanasia were pre-approved by the University of Cincinnati Animal Care and Use Committee. Any surviving animals at the end of the study were subjected to anesthesia by carbon dioxide application, immediately followed by a confirmatory cervical dislocation for euthanasia and the terminal tissue collection. All efforts were made to minimize suffering. An independent review and approval of our cell and virus methods was conducted by the Institutional Biosafety Committee (IBC).

### Cell culture

Mouse iPSC were generated and maintained in Dulbecco's Modified Eagle's medium (DMEM) with 1000 IU/ml leukemia inhibitory factor (LIF, Chemicon, ESGRO), as described previously [11]. Mouse embryonic fibroblasts (MEF) obtained from embryos at 14 days post-coitum were prepared and treated with mitomycin-C (10 µg/mL) to control MEF overpopulation. Embryoid body (EB) formation was promoted by placing iPSC in 25 µl hanging drops (∼250 cells per drop) and culturing the suspension in iPSC medium without LIF. After 5 days, EBs were transferred to 0.1% gelatin-coated dishes. Medium was changed the following day and then changed every other day to maintain viable cells. Cells were then allowed to differentiate into large aggregates, using differentiation media and growth factor conditions reported previously by Stevens et al. [12] to induce differentiation into CM. Cell culture conditions were further modified to optimize directed differentiation into precursor CM with lentiviral genomic manipulation.

### Construction and production of lentiviral vectors

CM precursors from iPSC were enriched by transducing with lentiviral vector encoding markers under the control of a CM-specific promoter (NCX1). The final construct containing CM-specific promoter (NCX1) driving firefly luciferase (pLVX-NCX1-Fluc-PuroR-IRES-ZsGreen1) is described in our earlier publication [13]. “Promoterless” pLVX-Fluc-IRES-ZsGreen1 vector constructs were generated as a control. In all cases, correct orientation of the insert was confirmed by sequencing. For viral particle production, HEK293FT cells (Invitrogen)were seeded on a 10 cm plate to obtain 70-80% confluence on the day of transfection. These cells were then transfected with plasmid vector using Lenti-X HT packing system (Clontech) following manufacturer's instructions. The crude viral suspension was harvested from HEK 293FT cell cultures 48 hours after transfection and filtered (0.45 µm) for use *in vitro*. Viral particles were stored at −80°C until use.

### Genetically selected CM and its purification

Lentivirus transduction medium was prepared by adding pLVX-NCX1-Fluc-PuroR-IRES-ZsGreen1 lentivirus (MOI∼35) and 8 µg/ml polybrene to each 3 ml of iPSC medium. EB were cultured on day-10 with transduction medium to determine the cardiac-specific expression of the NCX1 promoter. To confirm the construction of an overexpressing NCX1 promoter iPSC cell line, iPSC (2.5×10^5^) were assayed using a FACS Calibur flow cytometer (Becton-Dickinson, Franklin Lakes, NJ) 5–7 days after transduction. The sorted GFP^+^ cells were plated into a 96-well plate pre-plated with MEF. Then, the single iPSC colonies were expanded into colonial cell lines and were tested individually for firefly luciferase (Fluc) activity during cardiac differentiation. Clones that showed stable Fluc activity were selected for further characterization and *in vivo* studies. To assure CM enrichment, continuous puromycin (1.5 µg/ml) conditions were used to reduce non-CM formation.

### Real-time PCR assay

Cells were collected for total RNA extraction later using TRIzol Reagent (Ambion, Life Technologies, Grand Island, NY). Total RNA was used for reverse transcription in a miScript II RT kit (Qiagen). qPCR was performed using miScript SYBR Green PCR kit (Qiagen, Valencia, CA). The expression of genes of interest was normalized to that of GAPDH. The primers for qPCR were listed as follows: **GATA4** forward primer: 5′- TCTGGCTGGCCGAGAGCAGT-3′, reverse primer: 5′-GGCTGTGCAGGA CTGGGCTG-3′; **Nkx2.5** forward primer: 5′- TGGGTCTCAATGCCTATGGCTACA-3′, reverse primer: 5′-GACGCCAAAGTTCACGAAGTTGCT-3′; **ACTC** forward primer: 5′-CCAGGATGTGTGACGACGAG-3′, reverse primer: 5′-TCCCATACCCACCA TGACAC-3′; **α-MHC** forward primer: 5′-TCTGCCTACCTTATGGGGCT-3′, reverse primer: 5′-ACTTGCTGTACACTCTGCCC-3′; **GAPDH** forward primer: 5′-CCAAG GCTGTGGGCAAGGTC-3′, reverse primer: 5′- GGCAGGTTTCTCCAGGCGG-3′.

### Preparation of monolayered cell sheet

Temperature responsive 35 mm UpCell dishes (CellSeed) were coated with 10% fetal bovine serum and incubated at 37°C overnight. Mouse embryonic fibroblasts (MEF) were seeded on the dish for 5 hours to form a cell substrate for iPSC-derived precursor CM. Then the above-treated iPSC derived CM (5×10^6^) with 3 ml of culture medium were added and continuously cultured for 24 h. Then the temperature of dishes was decreased from at 37°C to 20°C. The iPSC-derived CM sheet detached within 30 minutes. Immediately after detachment, the CM sheet was gently transferred onto the epicardial surface of the infarcted area of the myocardium as described previously [14].

### Myocardial infarction model and iPS-derived CM sheet transplantation

A transmural regional MI model was generated by left anterior descending coronary artery ligation in C57BL/6 female mice (8–10 weeks) under general anesthesia (0.01 mL/g of a solution containing ketamine 10 mg/mL, xylazine 2 mg/mL by intraperitoneal injection), as described previously [15]. Animals were mechanically ventilated using a rodent ventilator (Model 845, Harvard Apparatus, South Natick, MA) connected to an endotracheal tube. The heart was exposed by a left side limited thoracotomy and the left anterior descending artery (LAD) was ligated with a 6-0 polyester suture 1 mm from tip of the normally positioned left auricle. Immediately after MI induction, an iPSC-derived monolayer CM sheet was apposed to the surface of LAD distribution overarching the MI area. The mice were randomly divided into four groups, as follows: 1) Sham operated mice had a loose suture placed around LAD (Sham group); 2) MI operated mice (MI group); 3) MI plus cell sheet without receiving NCX1 promoter (MI + iPSC^Null^ group), and 4) MI plus cell sheet with receiving NCX1 (MI + iPSC^NCX1^).

### Scanning electron microscopic and transmission electron microscopic images

For scanning electron microscope (SEM) observation, heart tissue specimens with the iPSC-CM sheet were fixed in 4% paraformaldehyde solution overnight and then coated with plasma for 2 minutes. Specimens were observed using a scanning electron microscope (XL 30-ESEM, Philips). For transmission electron microscope (TEM) observation, heart tissue with the iPSC-CM sheet was fixed in 2.5% glutaraldehyde overnight then incubated while protected from light in 1% osmium tetroxide for 2 hours. After washing in distilled water, specimens were incubated in 2% uranyl acetate for 2 hours at room temperature and then dehydrated in graded ethanol concentrations. Finally, samples were embedded in molds with fresh resin. Ultrathin sections (70 nm) were examined in detail using an electron microscope (JEM, JEOL-USA).

### Analysis of left ventricular (LV) fibrosis and anterior wall thickness

Fixed heart tissue was embedded in paraffin and LV minor axis cross-sections from mid-LV to apex were stained with Masson's Trichrome. These sections were used to quantify LV infarct size in all treatment groups. An Olympus BX41 microscope equipped with CCD (Magna-Fire TM, Olympus) camera captured LV area images from each slide. The LV infarct area and total LV area of each image as well as left ventricular anterior wall thickness (region of infarct) were measured using Image-Pro-Plus software (Media Cybernetics Inc., Carlsbad, CA, USA). The infarct (fibrosis) area was calculated as a percent of the total LV area (infarct area/total LV area) ×100.

### Measurements of left ventricular function

Cardiac function was assessed using transthoracic echocardiography (iE33 Philips) with a 15-MHz probe. Echocardiography was performed with animals under light general anesthesia at 1 day before and at 4 weeks after cell sheet implantation to assess systolic and diastolic dimensions of the LV. Hearts were imaged in 2-D long-axis view at the level of the greatest LV diameter as described previously [15]. LV ejection fraction index (EF) was calculated as: EF (%)  =  [LV end-diastolic dimension cubed (LVDd)^3^ minus LV end systolic dimension cubed (LVDs)^3^/(LVDd)^3^] ×100. LV minor axis fractional shortening (FS) was determined as [(LVDd–LVDs)/LVDd] ×100. All measurements were performed according to the American Society for Echocardiology leading-edge technique standards, and were averaged over three consecutive cardiac cycles.

### Statistical analysis

Statistical comparison of data was performed using unpaired two-sided *t*-tests. One-way analysis of variance was used for multiple group comparisons. Differences were considered significant at values of *p*<0.05. All values are expressed as mean ± S.E.M.

## Results

### Inducing high purified CM from iPSC and generating a monolayer cell sheet

**Figure 1:**
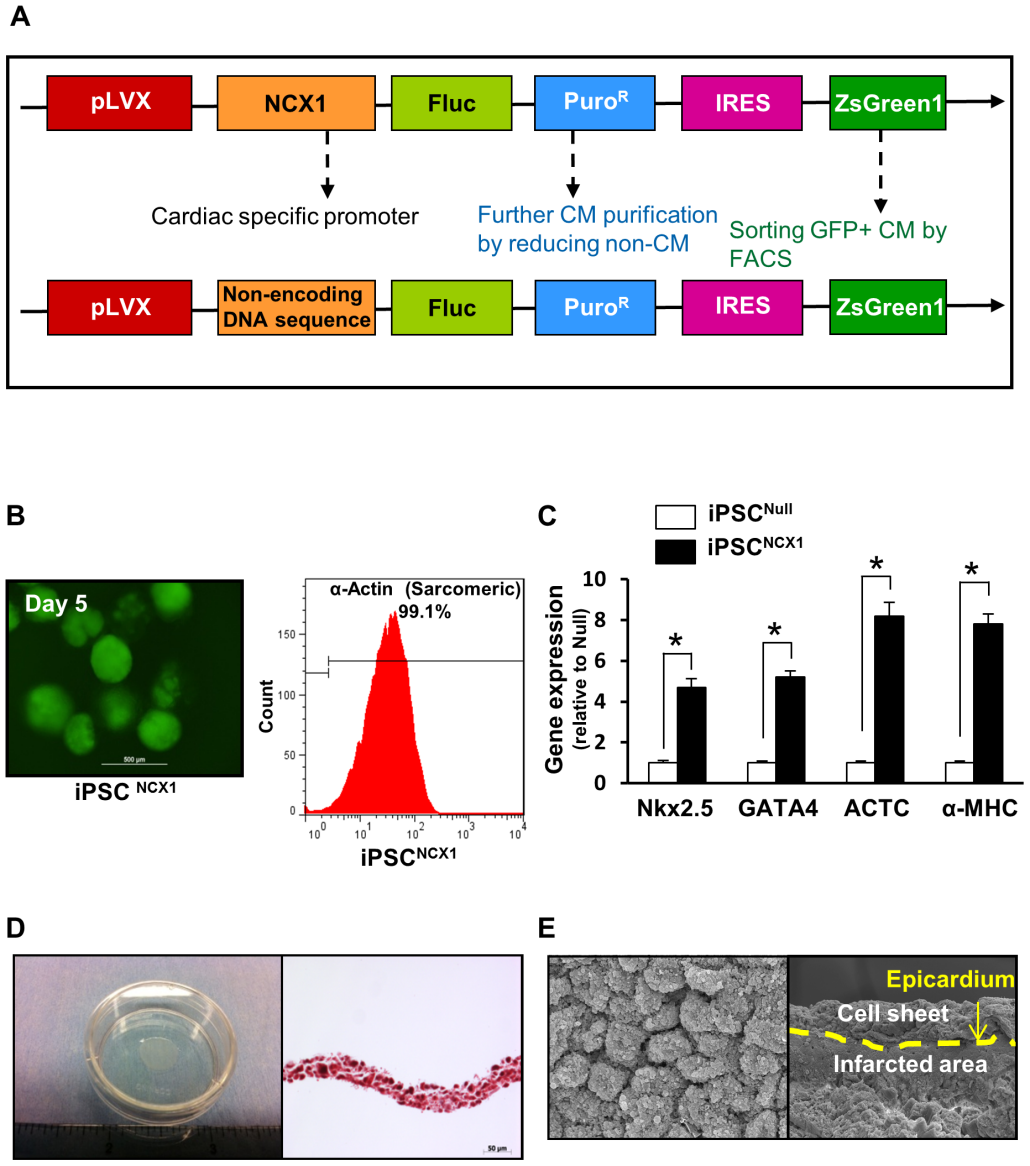
iPSC derived CM and features of cell sheet. **A**: Construction of lentiviral vector expressing ZsGreen1 (green fluorescence) under the control of a CM-specific promoter (NCX1). The final construct contained the CM-specific promoter (NCX1) driving firefly luciferase (pLVX-NCX1-Fluc-PuroR-IRES-ZsGreen1). NCX1  =  sodium-calcium exchanger promoter. Fluc  =  firefly luciferase; **B**: EB from iPSC on day 5 were used to determine efficiency of NCX1/GFP on iPSC-derived CM selection under immunofluorescent microscopy (Left panel). On Day 15 differentiated CM cells were analyzed by flow cytometry selecting for α-actinin positive cells (Right panel). **C**: The cardiac gene expression profile in iPSC-derived CM was assessed by qPCR. Quantitative data for Nkx2.5, GATA4, ACTC, and α-MHC expression at day 15 after iPSC differentiation. All values expressed as mean ± SEM. n = 6 for each group. **D**: iPSC-CM sheet was cultured on temperature-responsive culture dishes and the cell sheets spontaneously detached as monolayer cell sheet when the temperature was lowered to 20°C for 30 min (Left). Cell sheets were approximately 10 mm in diameter (Left) with a 30 µm thickness (Right). **E**: Scanning electron microscopic image of iPSC-CM sheet (Left) and ischemic heart tissue 4 weeks after implantation of iPSC-CM sheet (Right).

The final construct containing the CM-specific promoter (NCX1) driving firefly luciferase (pLVX-NCX1-Fluc-PuroR-IRES-ZsGreen1) was used to generate iPSC-derived CM. “Promoterless” pLVX-Fluc-IRES-ZsGreen1 vector constructs were generated and then served as control (Figure 1A). EB derived from iPSC were observed using an immunofluorescent microscope 5 days after they were transduced with NCX1 promoter guided pLVX-NCX1-Fluc-PuroR-IRES-ZsGreen1 (GFP) lentivirus vectors (Figure 1B, left panel). Following that treatment, α-sarcomeric actin (a CM-specific bio-marker) positive cells were obtained by cell sorting the iPSC^NCX1^ group (Figure 1B, right panel) as compared with the iPSC^Null^ group. In addition, cardiac gene expression levels of Nkx2.5, GATA4, ACTC, and α-MHC in iPSC^NCX1^ were upregulated significantly at day 15 after iPSC differentiation as compared with the iPSC^Null^ group (Figure 1C).

The above treated iPSC-derived CM with 3 ml of culture medium were seeded onto 3.5-cm temperature-responsive dishes pre-coated with an MEF substrate and incubated to confluence at 37°C for 48 h. The culture dishes were then incubated at 20°C for 30 minutes, where upon the iPSC-CM cell sheet detached spontaneously (Figure 1D, left panel). The thickness of the cell sheet was approximately 30 µm (Figure 1D, right panel). Scanning electron microscope was used to obtain a surface view of the cell sheet and confirm that each CM was connected very tightly after cell sheet detachment and before cell sheet implantation (Figure 1E, left panel) and that the cell sheet was in close contact with the epicardium of the infarcted heart tissue 4 weeks after the iPSC-CM sheet implantation (Figure 1E, right panel).

### Ultrastructural features of iPSC-CM sheets

**Figure 2:**
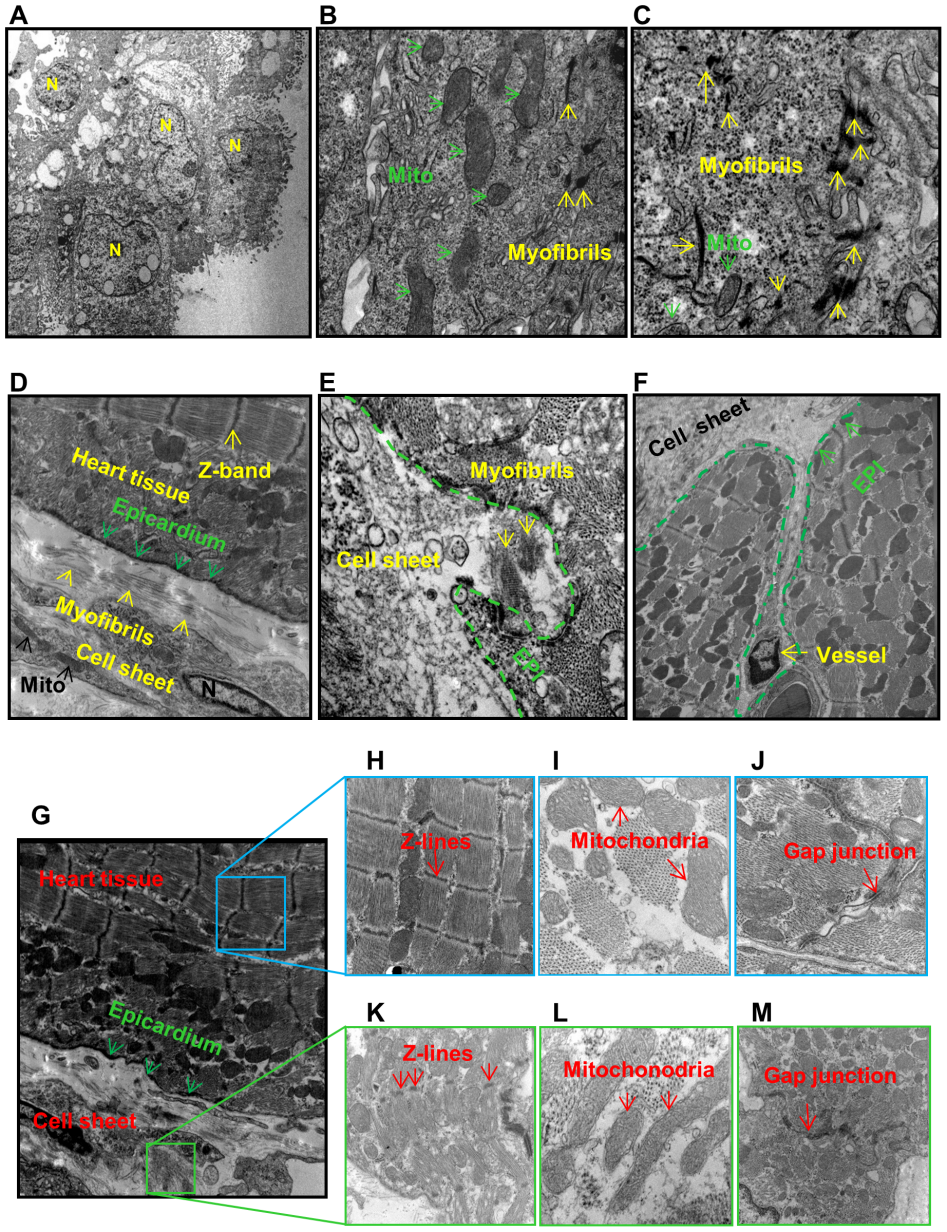
Ultrastructural features of iPSC-CM sheets in repairing MI. **A–C**: Ultrastructural features of the cell sheet in culture dishes were examined using transmission electron microscope. Cell sheet is located in the lower area. N =  nuclei; Mito  =  mitochondria; EPI  =  epicardium. **D–F**: iPSC-derived CM and cell sheets in infarcted myocardium 4 weeks after cell sheet implantation. **G**: Cell sheets in infarcted myocardium 4 weeks after cell sheet implantation. **H–J**: MI border zone 4 weeks after cell sheet implantation. **K–M**: Features of cell sheet implanted on the surface of infarcted myocardium.

Ultrastructural features of CM precursors in the cell sheet were examined in culture dishes and documented using transmission electron microscope (Figure 2). These features include nuclei (N), myofibrils (yellow arrowheads), mitochondria (Mito), and Z-line and gap junctions (Figure 2A-C).

Four weeks after cell sheet implantation, the cell sheet was tightly connected with epicardium (Figure 2D) and some parts of the cell sheet had begun to invade the infarcted area of myocardium. This was associated with newly formed myofilaments (Figure 2E, yellow arrowheads) and blood vessels (Figure 2F) in iPSC^NCX1^ group. In contrast with adult mature myofibrils (Figure 2H), mitochondria (Figure 2I), and gap junctions (Figure 2J) in border areas (Figure 2H–J), the size, shape and arrangement of myofibrils (Figure 2K), mitochondria (Figure 2L), and gap junctions (Figure 2M) in cell sheet were irregular and immature. Myofibrils with Z-bands were evident in the cytoplasm of the iPSC-derived CM, indicating the formation of sarcomeres (Figure 2K). iPSC-CM showed myocardial ultrastructural features of early and an immature cardiac muscle cell phenotype containing myofilaments, large glycogen deposits, mitochondria, rough endoplasmic reticulum, and sarcoplasmic reticulum (Figure 2K–M). Noteworthy, gap junctions were formed by desmosomes and fascia adherens.

### Cell sheet attenuated heart remodeling and infarction size

**Figure 3:**
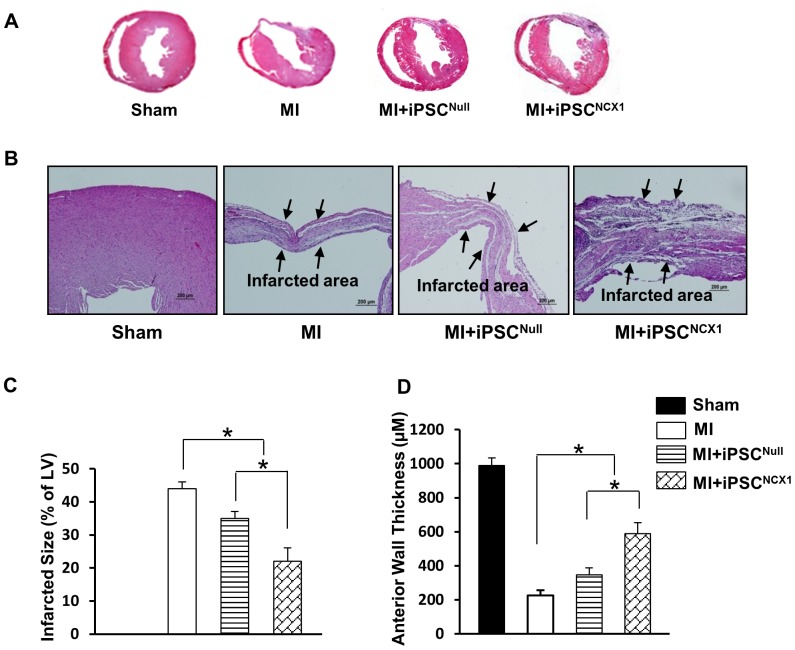
Cell sheet attenuated heart remodeling and infarction size. **A**: Left ventricular sections from all treatment groups (Masson's Trichome stained). **B**: Left ventricular anterior wall thickness was assessed. **C**: Percentage of infarcted area in hearts was analyzed at 4 weeks after cell sheet implantation and various treatments. All values were expressed as mean ± SEM. *p<0.05. *n* = 6 in each group. **D**: Left ventricular anterior wall thickness was measured and presented in the form of a bar diagram. All values were expressed as mean ± SEM. *p<0.05. n = 6 in each group. **Sham group**: Sham operated rats had a loose suture placed around the left anterior descending coronary artery (LAD); **MI group**: indicates myocardial infarction; **MI + iPSC^Null^ group**: MI plus cell sheet without receiving NCX1 promoter; **MI + iPSC^NCX1^ group**: MI plus cell sheet receiving NCX1 promoter.

Four weeks after cell sheet implantation, the pathological changes in the infarcted area and the LV anterior wall thickness (AWT) were assessed using Masson's Trichrome staining in all treatment groups (Figure 3A–D). LV infarct size and a percent of total LV area was reduced significantly in the MI + iPSC^NCX1^ group (22.1±4.1%), and to a lesser degree in the MI + iPSC^Null^ group (35.5±2.1%) as compared with the untreated MI group (44.2±2.1%) (Figure 3B and C). LV AWT (Figure 3D) was significantly increased in the MI + iPSC^NCX1^ group (590.2±58.2 µm), and less so in the MI + iPSC^Null^ (347.1±28.1 µm) as compared with the untreated MI control group (227.1±26.2 µm).

### Assessment of heart function

**Figure 4:**
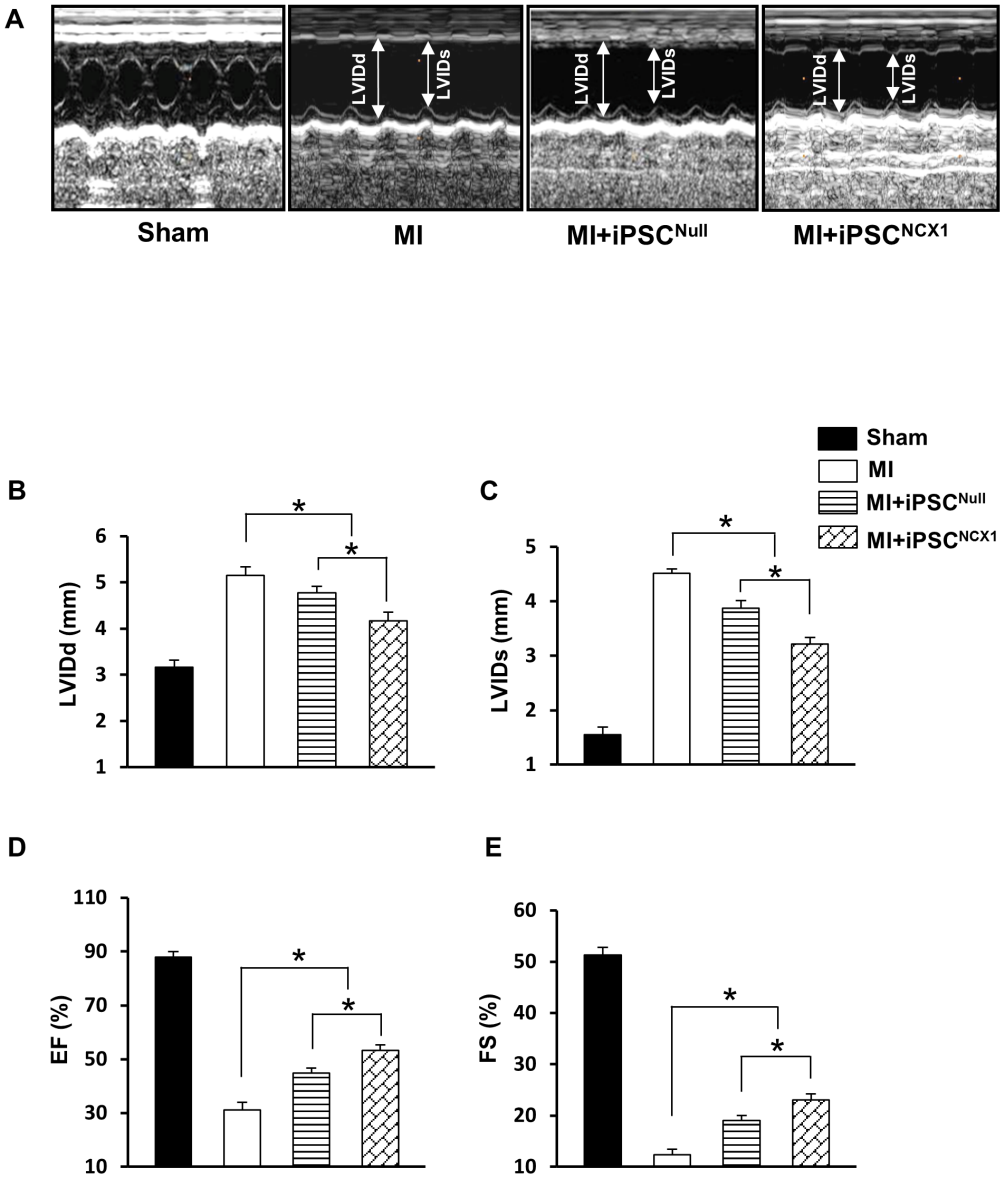
Effect of cell sheet on cardiac function and heart remodeling. **A**: M-mode echocardiograms in various treatment groups at 4 weeks after cell sheet implantation. (**B–E**): Quantification analysis for LVDd, LVDs, EF, and FS. LVDd: left ventricular end-diastolic dimension; LVDs: Left ventricular end-systolic dimension; EF: ejection fraction; FS: fractional shortening. * *p*<0.05. All values are expressed as mean ± SEM. (n = 5). **Sham group**: Sham operated rats had a loose suture placed around the left anterior descending coronary artery (LAD); **MI group**: indicates myocardial infarction; **MI + iPSC^Null^ group**: MI plus cell sheet without receiving NCX1 promoter; **MI + iPSC^NCX1^ group**: MI plus cell sheet receiving NCX1 promoter.

Echocardiography was performed on all subjects in the various treatment groups 4 weeks after cell sheet implantation (Figure 4). There were no significant differences in left ventricular function as indicated by left ventricular end-diastolic (LVDd), end-systolic diameters (LVDs), left ventricular ejection fraction (EF), fractional shortening (FS), and heart rate in all groups measured 1 day before or after MI induction by LAD occlusions (data not shown). Four weeks after MI and cell sheet application, both the MI + iPSC^NCX1^ and MI + iPSC^Null^ groups showed significant improvement in LVDd, LVDs, FS, and EF as compared with the MI control group (Figure 4). The most significant changes were seen in the MI + iPSC^NCX1^ group, where minor axis LVDd (4.1±0.2 mm) and LVDs (3.2±0.1 mm) were restored toward normal values, while both LV functions - EF (53.2±1.1%) and FS (23.1±0.5%) - were improved significantly as compared with the MI + iPSC^Null^ group (Figure 4B–E).

## Discussion

Despite recent advances in pharmacological and surgical approaches in the treatment of injured ischemic myocardium, mortality and morbidity following heart failure remains high. Moreover, conventional therapeutic options for treatment of MI are limited to preventing the progression of ventricular remodeling and delaying the onset of congestive heart failure [16].

iPSC have the potential to generate CM, and others and we contend that this approach holds significant promise for application in regenerative medicine as a potential treatment in patients with MI and heart failure. iPSC-derived CM sheet technology offers an attractive method for replacement of dead or damaged myocardium after MI. While there are two pre-eminent current methods of cell delivery in use today - intravascular injection and direct tissue injection [13]- the delivery of such grafted cell populations to the target tissues is uncertain using these methods. We proposed that this major therapeutic obstacle may be overcome by direct application of gene manipulated cell sheets using a minimally invasive surgical procedure to provide a supportive microenvironment for cell engraftment, migration, and survival at the tissue site for which therapy is most needed. This recommendation is supported by the major findings of this study: **1**) genetically manipulated iPSC with the cardiac-specific NCX1 promoter significantly enhanced CM differentiation, **2**) morphologic features of CM derived from iPSC in cell sheets are significantly different in size and shape as compared adult mature CM in heart tissues, **3**) transplanted iPSC-CM are good candidates for treatment of MI and myocardial tissue regeneration in terms of engraftment properties and overall CM survival, and **4**) iPSC-derived CM sheets applied to an ischemic region of the heart attenuate ventricular remodeling of post-MI tissue as evidenced by an increased anterior LV anterior wall thickness, reduced infarct size, and consequent restoration of LV function.

Purification of specific cell lineages derived from iPSC is vitally important for prevention of teratoma formation [13]. We used several steps to avoid teratoma formation. First, lentiviral vectors containing specific cardiac promoters guide differentiation of progenitor cells exclusively into CM and these vectors also expressed both GFP and puromycin resistance (Puro^R^). GFP was used as a marker for further purification of CM and their precursors by FACS. Continuous selection of CM in puromycin (1.5 µg/ml) enriched media was used to further reduce potential contamination by non-CM. These methods proved most effective, eliminating teratoma formation during the 4 weeks of this study.

## Conclusions

Manipulation of iPSC with lentivirus vectors encoding markers under the control of NCX1 promoter yields a large number of highly pure CM, and grafting of iPSC-derived CM on cell sheets over scarred areas of myocardium subsequent to coronary artery ligation reduced ventricular remodeling and LV fibrosis subsequent to new CM populations and new blood vessel formation, leading to enhanced restoration of cardiac function after MI.
